# Infectious bronchitis corona virus establishes productive infection in avian macrophages interfering with selected antimicrobial functions

**DOI:** 10.1371/journal.pone.0181801

**Published:** 2017-08-01

**Authors:** Aruna Amarasinghe, Mohamed Sarjoon Abdul-Cader, Sadiya Nazir, Upasama De Silva Senapathi, Frank van der Meer, Susan Catherine Cork, Susantha Gomis, Mohamed Faizal Abdul-Careem

**Affiliations:** 1 Department of Ecosystem and Public Health, Faculty of Veterinary Medicine, University of Calgary, Health Research Innovation Center 2C53, Calgary, Alberta, Canada; 2 Department of Veterinary Pathology, Western College of Veterinary Medicine, University of Saskatchewan, Saskatoon, Saskatoon, Canada; Sun Yat-Sen University, CHINA

## Abstract

Infectious bronchitis virus (IBV) causes respiratory disease leading to loss of egg and meat production in chickens. Although it is known that macrophage numbers are elevated in the respiratory tract of IBV infected chickens, the role played by macrophages in IBV infection, particularly as a target cell for viral replication, is unknown. In this study, first, we investigated the ability of IBV to establish productive replication in macrophages in lungs and trachea *in vivo* and in macrophage cell cultures *in vitro* using two pathogenic IBV strains. Using a double immunofluorescent technique, we observed that both IBV Massachusetts-type 41 (M41) and Connecticut A5968 (Conn A5968) strains replicate in avian macrophages at a low level *in vivo*. This *in vivo* observation was substantiated by demonstrating IBV antigens in macrophages following *in vitro* IBV infection. Further, IBV productive infection in macrophages was confirmed by demonstrating corona viral particles in macrophages and IBV ribonucleic acid (RNA) in culture supernatants. Evaluation of the functions of macrophages following infection of macrophages with IBV M41 and Conn A5968 strains revealed that the production of antimicrobial molecule, nitric oxide (NO) is inhibited. It was also noted that replication of IBV M41 and Conn A5968 strains in macrophages does not interfere with the induction of type 1 IFN activity by macrophages. In conclusion, both M41 and Con A5968 IBV strains infect macrophages *in vivo* and *in vitro* resulting productive replications. During the replication of IBV in macrophages, their ability to produce NO can be affected without affecting the ability to induce type 1 IFN activity. Further studies are warranted to uncover the significance of macrophage infection of IBV in the pathogenesis of IBV infection in chickens.

## Introduction

Infectious bronchitis (IB) is primarily a respiratory disease of chickens but with potential to cause more widespread infection in the urinary and reproductive tracts in chicken leading to significant production losses in commercial broiler and layer flocks worldwide [1]. The causative infectious bronchitis virus (IBV) belongs to the family Coronaviridae. The disease is usually characterized by high morbidity and low mortality in mature birds, whereas in naive young birds (2–3 weeks of age), mortality up to 100% can be observed [1]. Being an RNA virus with the ability to mutate and recombine, IBV persist as numerous serotypes and strains. The control of IB relies on vaccination. Vaccines are available for commonly occurring serotypes and strains but they are not necessarily antigenically similar to the wild-type viral strains circulating in poultry barns. Although, these vaccine strains may provide some degree of protection for some related strains known as protectotypes [2], the commonly available vaccines may not elicit protective immune responses in a flock if the field strains are antigenically very different from the vaccine strains. Owing to this reason, vaccination against IBV is not currently considered to be a very effective control method and other biosecurity measures are necessary to prevent the introduction of IBV into poultry production facilities.

IBV is known to replicate in the respiratory tract leading to changes in the muco-cilliary clearance mechanism, as such, expose the IBV infected birds to secondary bacterial infections. Additionally, IBV has tropisms for a variety of tissues. However, the mode of dissemination from the common route of entry, i.e. the respiratory route, to the rest of the body systems could potentially be due to the initial viremia [3]. Once disseminated, IBV infects epithelial cells of the reproductive and urinary systems, particularly the oviduct and kidney depending on the infecting strain [4]. Recently, it has been shown that a nephro-pathogenic strain of IBV (B1648) could replicate in peripheral blood monocytes leading to viremia [4]. The infection of circulating monocytes could potentially disseminate IBV to the urinary tract, liver and spleen [4].

Macrophages play roles in innate immune responses, as well as in mounting adaptive immune responses by functioning as antigen presenting cells, as such they are critical in protecting animals from microbial infections. Although it is known that macrophage numbers are elevated in the respiratory tract in response to IBV infection [5], the role played by macrophages in IBV infection, particularly if they serve as a target cell for viral replication is not known. Macrophages have been implicated to play in an important role in the pathogenesis of some animal and human viruses including Marek’s disease virus in birds [6], feline corona virus in cats [7], and human immunodeficiency virus (HIV) [8]. It was also shown that coronaviruses such as severe acute respiratory syndrome (SARS)-coronavirus (CoV) can replicate within human macrophages [9] thereby interfering with macrophage functions leading to severe pathology [10]. However, a single report based on *in vitro* studies indicated that IBV, particularly nonpathogenic Beaudette and Massachusetts type 82822 strains do not replicate in avian macrophages [11].

Therefore, in this study we investigated the interaction of IBV with macrophages in lungs and trachea *in vivo* and macrophage cell cultures *in vitro* using two IBV strains, Connecticut A5968 (Conn A5968) and Massachusetts-type 41 (M41) which are known to induce clinical disease and pathological lesions in chickens. As implicated in some other viruses, we hypothesized that these two strains of IBV replicate within avian macrophages leading to productive replication and interfering with selected macrophage functions in the process.

## Materials and methods

### Animals, virus and cells

The Veterinary Science Animal Care Committee (VSACC) and Health Science Animal Care Committee (HSACC) have approved the use of specific pathogen free (SPF) chickens used in all our experimental procedures. One day old unsexed SPF layer chickens (White Leghorn) were obtained from the Canadian Food Inspection Agency (CFIA), Ottawa, and raised to six days of age for use in these experiments. These chickens were not immunized and were housed in high containment poultry isolators at the University of Calgary’s Spyhill campus or at the Foothills campus, with ample access to a standard food ration and water.

Conn A5968 and M41 strains of IBV as well as avian fibroblast cells line (DF-1) were purchased from American Type Culture Collection (ATCC, Manassas, Virginia, United States). The macrophage cell line, Muquarrab Qureshi-North Carolina State University (MQ-NCSU), which is derived from mononuclear cells harvested from the spleen of a chicken [12] was kindly gifted by Dr. Shayan Sharif (University of Guelph, Canada). MQ-NCSU cells were maintained in LM-HAHN media and fungizone (250μg/ml) at 40°C and 5% CO_2_ [13]. The vesicular stomatitis virus tagged with green fluorescent protein (VSV-GFP) was obtained from Dr. Markus Czub, University of Calgary, Canada.

### Virus propagation and titration

IBV M41 and Conn A5968 strains were propagated in 9-day old SPF embryonated eggs and allantoic fluid harvested at 3 days post-infection (dpi). Viral titers were determined using 9 days old SPF embryonated chicken eggs and expressed as fifty percent embryo infectious dose (EID_50_). Briefly, ten-fold serial dilutions of virus were prepared in PBS (1 × Dulbecco’s phosphate buffered saline (DPBS) (Gibco, Life Technologies, Burlington, ON, Canada) and 100 μL of the dilutions were inoculated into the allantoic cavity of eggs. The inoculated eggs were incubated at 37.2°C and relative humidity of 60% for another 6 days. On 6 dpi, the embryos were euthanised and examined for the presence of embryo dwarfing and curling, which is characteristic for IBV infection. Based on these observations, end points were determined [14, 15]. Virus titers were assessed by the fifty percent end-point, according to the method described by Reed and Muench, and expressed as EID_50_/mL.

### Experimental design

#### Demonstration of IBV antigens in macrophages in IBV infected chickens *in vivo*

Six day old SPF chickens were infected intra-tracheally with either M41 (n = 5) or Conn A5968 (n = 5) strains of IBV at the dose rate of 2.75 x 10^4^ EID_50_/bird [5], 4 birds were treated with PBS as uninfected controls. Upon infection, the experimental chickens were monitored for development of specific and non-specific clinical signs such as huddling under the lamp, ruffled feathers, droopy wings and increased respiratory rates. At 4 dpi, trachea and lung samples were collected into Optimum Cutting Media (OCT) (VWR, Mississauga, Ontario, Canada) for preservation in -80°C with a view of using for staining of macrophages and IBV antigens. In addition, samples of trachea and lungs were fixed in 10% formalin for histological examination. The establishment of IBV infection in the infected animals was confirmed by IBV antigen detection using immunofluorescent technique along with characteristic histological changes in the trachea and lungs.

#### *In vitro* experiments

**Infectability of avian macrophages with IBV *in vitro*:** Macrophages (MQ-NCSU) were cultured on coverslips placed in 6-well plates (1.5x10^6^ viable cells per well). After 24 hours cells were washed twice with Hank’s balanced salt solution (HBSS) and infected with 0.1 multiplicity of infection (MOI) of IBV M41 or Conn A5968 strains. After one hour, virus inoculum was removed, cells were washed twice with HBSS and supplied with fresh medium. At 24 hours post-infection (hpi), each section was subjected to two-color immunostaining to co-localize macrophages and IBV antigens. Each experiment was performed in triplicate including uninfected controls.

**Infectability of avian macrophages with IBV *in vitro* leading to productive infection:** Macrophages (MQ-NCSU cells) were suspended in LM-HAHN media and seeded in 6-well plates at a density of 1.5x10^6^ cells per well and incubated at 37°C in 5% CO_2_ for 24 hours. The confluent monolayers were washed twice with HBSS and infected with IBV M41 or Conn A5968 strains at an MOI of 0.1 or mock infected in triplicate. At one hour, the inoculum was completely removed, the cells were washed twice with HBSS in order to remove potential residual inoculum, and incubated with serum-free 2X MEM media (Gibco Life Technologies, Burlington, ON, Canada) at 37°C, 5% CO_2_. At 6, 12, 18, 24 and 48 hpi, cell culture supernatants derived RNA was used to quantify IBV RNA following IBV infection of macrophages. The experiment was repeated three times.

**Detection of IBV particles in avian macrophages:** Macrophages (MQ-NCSU cells) were cultured on T25 culture flasks until 90% confluence was achieved. After 24 hours, the cells were washed twice with HBSS and infected at an MOI of 0.1 with IBV M41 or Conn A5968 strains or mock infected. Each experiment was performed in triplicate. After one hour, the plates were washed twice with HBSS and incubated with serum-free 2X MEM media for 24 hours at 37°C and 5% CO_2_ before harvesting cells for transmission electron microscopy (TEM) imaging.

**IBV interference with nitric oxide (NO) production and type 1 interferon (IFN) activity during the infection of macrophages:** To determine whether type 1 IFN activity or NO production is induced in macrophages following IBV infection *in vitro*, MQ-NCSU cells were seeded in 12-well plates at 0.75 x 10^6^ cells per well and incubated for 24 hours. Subsequently, they were either infected or treated with 0.1 MOI of IBV M41 strain, 0.1 MOI of IBV Conn A5968 strain, 1μg/ml lipopolysaccharide (LPS) (SIGMA, Saint Louis, Missouri, USA) as a positive control for NO induction or double stranded (ds) RNA (Invivogen, San Diego, California, USA) 50μg/ml as a positive control for type 1 IFN induction. RPMI media was used as the negative control. All experiments were done in triplicates and repeated three times. Twenty-four hpi or treatment, culture supernatants were preserved at -80°C for subsequent quantification of NO production or type 1 IFN activity.

### Techniques

#### Histology

The Diagnostic Services Unit of the University of Calgary Faculty of Veterinary Medicine processed the tissues kept in 10% formalin and provided with us with stained hematoxylin and eosin (H&E) sections for evaluation.

#### RNA extraction and complementary DNA (cDNA) synthesis

Trizol LS reagent (Ambion, Invitrogen Canada Inc., Burlington, ON, Canada) was used for RNA extraction from 250 μl of macrophage-culture supernatants following manufacturer’s instructions. The RNA pellet was re-suspended in 20 μl of RNase-free water. The RNA concentration was quantified using the Nanodrop 1000 spectrophotometer at 260 nm wavelength (Thermo Scientific, Wilmington, DE, USA). The RNA (2000 ng) was transcribed using 10X RT random primers (High Capacity cDNA Reverse Transcription Kit (Invitrogen Life Technologies, Carlsbad, CA) as has been instructed by the manufacturer.

#### IBV RNA quantification

A quantitative PCR (qPCR) protocol was used for IBV RNA determination in the cell culture supernatants using forward primer, IBV N Fw: 5’GACGGAGGACCTGATGGTAA3’ and reverse primer, IBV-N Re: 5’CCCTTCTTCTGCTGATCCTG3’ [5]. All qPCR assays were conducted in a CFX96 Real-Time System C1000 Thermal Cycler (Bio-Rad Laboratories, Mississauga, ON) using 96-well un-skirted, low profile PCR plates (VWR, Edmonton, AB, Canada) and sealed with MicroAmp Optical Adhesive Films (Applied Biosystems, Foster City, California, USA). Fast SYBR^®^ Green Master Mix (Invitrogen, Burlington, ON, Canada) with 5 nM of each of the gene-specific primers, 200 ng of cDNA derived from each sample and RNAse-free water were used in the reaction in a final reaction volume of 20 μL. A positive control (plasmid) and negative template control (NTC) (RNAse-free water) and no reverse transcriptase control (NRT), and the dilution series of the N-gene plasmid as previous described [5] were included. Thermal cycling parameters were 95°C for 20 seconds; 40 cycles of amplification/extension at 95°C for 3 seconds, and 60°C for 30 seconds followed by a melting curve analysis at 95°C for 10 seconds (Segment 1), 65°C for 5 seconds (Segment 2) and 9°C for 5 seconds (Segment 3). Fluorescent acquisition was done at 60°C for 30 seconds. Amplification efficiency was determined by running a standard curve over a linear dynamic range (LDR) from 7 log_10_ copies/reaction to 1 log_10_ copies/reaction in the standard curve.

#### Immunofluorescent assay

**Double immunostaining of cryosections:** Frozen tissue sections (5 μm) from OCT preserved tissues were adhered on to positively charged slides, fixed with acetone (4°C) and stained for macrophages and IBV antigens sequentially. After protein blocking with 5% goat serum, the cryosections were incubated for 30 minutes with mouse monoclonal anti-chicken macrophage (KUL01) antibodies (Southern Biotech, Birmingham, Alabama, USA) (1:200 in PBS containing 5% goat serum) and washed three times after incubation. These sections were then stained with the secondary antibody, DyLight^®^ 550 conjugated goat anti-mouse IgG (H+L) (Vector Laboratories, Inc., Burlingame, California, USA) (1:500 in PBS containing 5% goat serum). Before being stained for IBV, the sections were blocked for endogenous avidin /biotin (Vector Laboratories, Burlingame, California, USA). A solution of 5% goat serum was used for 30 minutes for blocking followed by 30 minutes incubation using anti-IBV rabbit polyclonal serum (1:3000) (Federal Research Institute for Animal Health, Greifswald-Insel Riems, Germany). Rabbit IgG was used as the isotype control (Vector Laboratories, Burlingame, California, USA) (1:500). Goat biotinylated anti-rabbit IgG (H+L) (1:400) (Vector Laboratories, Burlingame, California, USA) was used as the secondary antibody. Avidin conjugated with Dylight^®^ 488 (Streptavidin) (Vector Laboratories, Burlingame, California, USA) was used for visualization. In the final step, the sections were mounted on Vectashield^®^ antifade mounting medium with 4′,6-Diamidine-2′-phenylindole dihydrochloride (DAPI) nuclear stain (Vector Laboratories, Burlingame, California, USA). All incubations were carried out at room temperature in a humidifying chamber to prevent drying the sections while on incubation.

**IBV antigen quantification in macrophages *in vitro*:** First, macrophages grown on coverslips were subjected to cell wall staining with wheat germ agglutinin conjugated with Alexa Fluor^®^ 594 (Invitrogen, Eugene, OR, USA) as has been instructed by the manufacturer. Then, the cells were fixed with 4% paraformaldehyde before being treated with 0.2% Triton X-100 (SIGMA, Saint Louis, Missouri, USA) for permeabilization. Avidin, biotin and protein blocking was followed by incubation with mouse anti-N monoclonal antibody (Vector Laboratories, Burlingame, California, USA) for 30 minutes (1:400) followed by goat anti-mouse IgG (H+L), which was conjugated to biotin (Vector Laboratories, Burlingame, California, USA) (1:400) for 20 minutes and by avidin conjugated with Dylight^®^ 488 (Streptavidin) (15 μg/ml) (Vector Laboratories, Burlingame, California, USA) for 20 minutes. Finally, coverslips were mounted on glass slides with Vectashield^®^ antifade mounting medium with DAPI nuclear stain (Vector Laboratories, Burlingame, California, USA) and sealed with lacquer.

#### Ultrastructural studies

Macrophages (MQ-NCSU cells) infected with M41 or Conn A5968 strains of IBV were harvested by scraping, centrifuged at 1000 g at 4°C for 10 minutes and submitted to the imaging facility of the University of Calgary for transmission electron microscope (TEM) imaging. For TEM, the cell pellets were immersed in 2% paraformaldehyde and 2.5% glutaraldehyde in 0.1M cacodylate buffer at pH 7.4 at 4°C for 2 hours. Then cells were embedded in Epon 812 mixture following treating the cells with 1% osmium tetroxide buffered with cacodylate and dehydrating *via* an acetone gradient. Sections of 60 nm were cut in a Leica EM UC7 (Leica IR, GmBH, Wetzlar, Germany) using a diamond knife. These sections were stained with 4% uranyl acetate and Reynolds’s lead citrate and analyzed with a Hitachi H7650 TEM at 80 kV. Images were obtained using an AMT 16000 digital camera. For obtaining high-resolution images, an EFI Tecnai F20 electron microscope was used.

#### NO production quantification in macrophage culture supernatants

The manufacturer’s instructions were followed for quantification of NO concentrations using the Griess assay reagent system (Promega Corporation, Madison, WI, USA). Briefly, cell culture supernatants were mixed with an equal volume of Griess assay reagent (1% sulfanilamide and 0.1% N-1-naftyletylendiamin dihydrochloride) in 96-well plates and incubated at room temperature for 10 minutes. The mean absorbance at 548nm was determined using SpectraMax M2 microplate reader (Molecular devices, Sunnyvale, Nova Scotia, Canada). Nitrite concentration of each sample was estimated based on sodium nitrate standard curve.

#### Type 1 IFN activity in macrophage culture supernatants

Culture supernatants of macrophages collected following IBV infection or treatment with dsRNA and LPS (250μl) were transferred to DF-1 cells grown for 24 hours. At 24 hours of culture supernatant transfer, these DF-1 cells were infected with VSV-GFP at an MOI of 0.1 and at 24 hpi the GFP signal was quantified under epifluorescent microscope following fixation with 4% paraformaldehyde and nuclear staining with Hoechst 33342 (Invitrogen, Eugene OR, USA).

#### Estimation of percentage of dead MQ-NCSU cells in culture

Following collection of MQ-NCSU culture supernatants, the cells were scraped into fresh media. The cell suspensions were centrifuged at 1000 rpm for 10 minutes at 4°C. The cell pellet was re-suspended in 100 μl of growth media and maintained in ice until counted. The number of live and dead cells in each of the suspension was enumerated using a hemocytometer after staining with Trypan blue. The number of dead cells in each well was presented as a percent of the total number of cells in the respective well.

### Data analyses

#### IBV genome load quantification

IBV RNA copies per 200 ng of cDNA were calculated following performing qPCR assay. A standard curve based on a serial dilution of plasmids was used for the calculation of IBV copies.

#### Quantification of macrophages and macrophages with IBV antigen

For the quantification of macrophage numbers, 5 areas (x40 magnification) with highest DyLight^®^ 550 signals were captured along with applicable nuclear stained (DAPI) areas. Similarly, for the quantification of IBV antigens, 5 areas (x40 magnification) with DyLight^®^ 488 fluorescent signals were captured along with applicable nuclear stained areas. The signals were captured using an Olympus IX51 inverted epifluorescent microscope and quantified using Image-J^®^ software (National Institute of Health, Bethesda, Maryland, USA).

#### Statistical analyses

Student’s t- test or one-way analysis of variance (ANOVA) test followed by Tukey’s were done using the Graphpad software (GraphPad Prism 5 Software, La Jolla, California, USA) for the confirmation of group differences. The differences were considered significant at P<0.05.

## Results

### Establishment of IBV M41 and Conn A5968 infections in the respiratory tract of chickens

**Fig 1:**
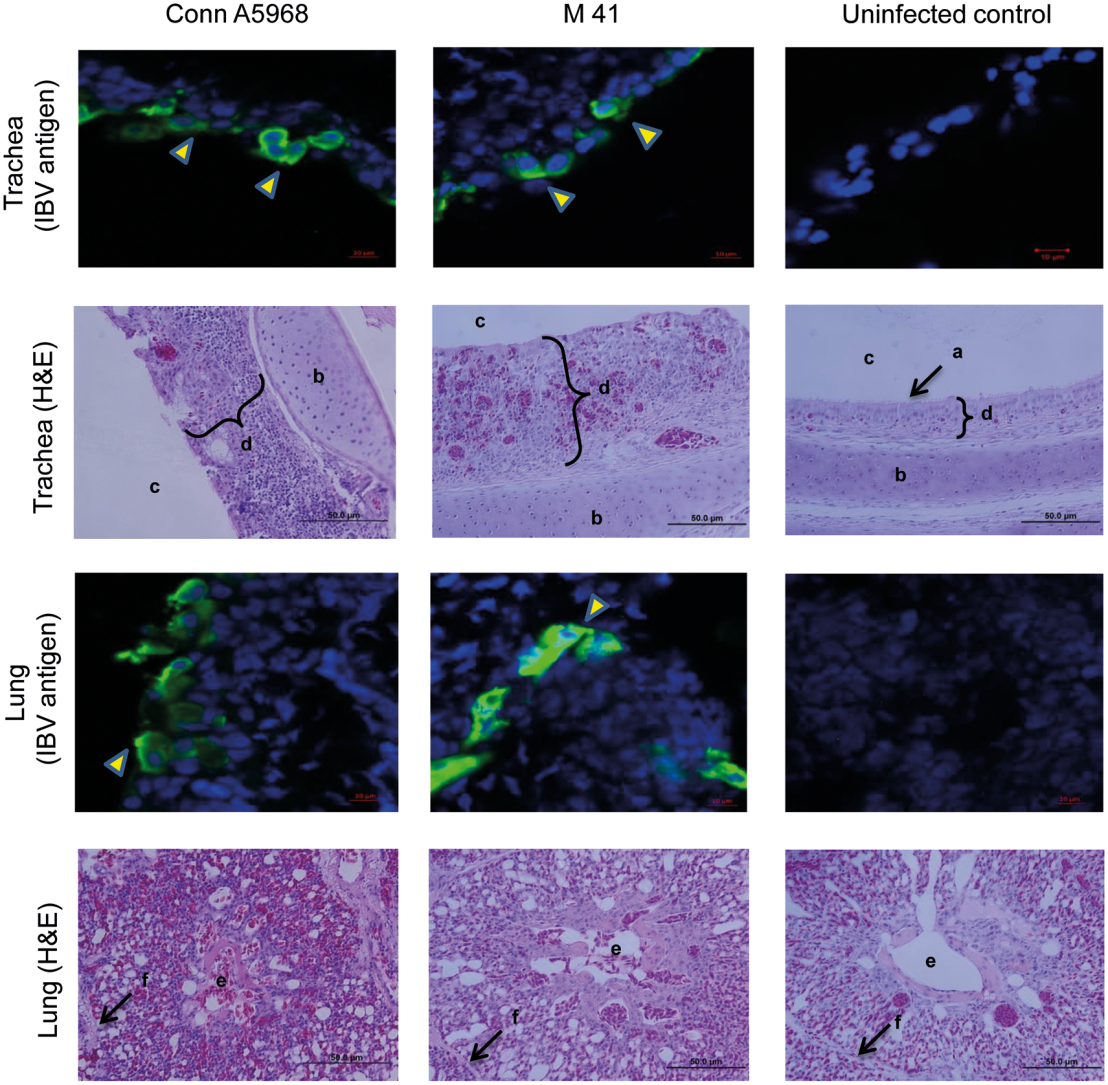
IBV M41 and Conn A5968 infections were evident by both IBV antigen demonstration and histological changes of the trachea and lungs. Six days old chickens were infected intra-tracheally with IBV M41 (n = 5) and Conn A5968 (n = 5) strains (2.75×10^4^ EID_50_ per bird) with uninfected controls (n = 4) and lung and tracheal tissues were collected in OCT and 10% formal saline at 4 dpi. Five μm frozen sections were immunostained for demonstrating IBV antigen and paraffin embedded sections were H&E stained. IBV N antigen in the tracheal and lung sections were stained using DyLight^®^ 550 fluorescent (arrow heads). In the IBV infected trachea, complete loss of cilia (a) and thickening of the mucosa with inflammatory cell infiltration can be seen compared to the control trachea. In the IBV infected lungs, inflammation leading to reduced air exchange areas and reduced lumen of parabronchi are visible. Also, the difference of mucosal thickening in the infected and uninfected trachea is shown (d). a = cilia, b = tracheal cartilage, c = tracheal lumen, d = mucosal layer, e = parabronchial lumen and f = interparabronchial septum.

Chickens infected with M41 or Conn A5968 strains of IBV showed transient and nonspecific clinical signs starting at 3 dpi that included huddling under the lamp, increased respiration and ruffled feathers. All the tracheal and lung sections of IBV Conn A5968 and M41 infected chickens collected on 4 dpi were positive for IBV antigen expression (Fig 1). Histological examination of the tracheas and lungs of IBV Conn A5968, M41 and control chickens revealed lesions suggestive of IBV infection in infected chickens but not control chickens (Fig 1). Lesions in IBV infected trachea consisted of deciliation of epithelial cells, mononuclear cell infiltration into underlying mucosa and metaplasia of epithelium from pseudo-stratified ciliated simple columnar to stratified squamous cells. The lesions in IBV infected lungs consisted of mononuclear cell infiltration reducing some air exchange areas of parabronchi and partial or complete occlusion of lumen of some parabronchi with inflammatory materials (Fig 1).

### Macrophage numbers in trachea and lungs following infection with IBV M41 and Conn A5968 strains

**Fig 2:**
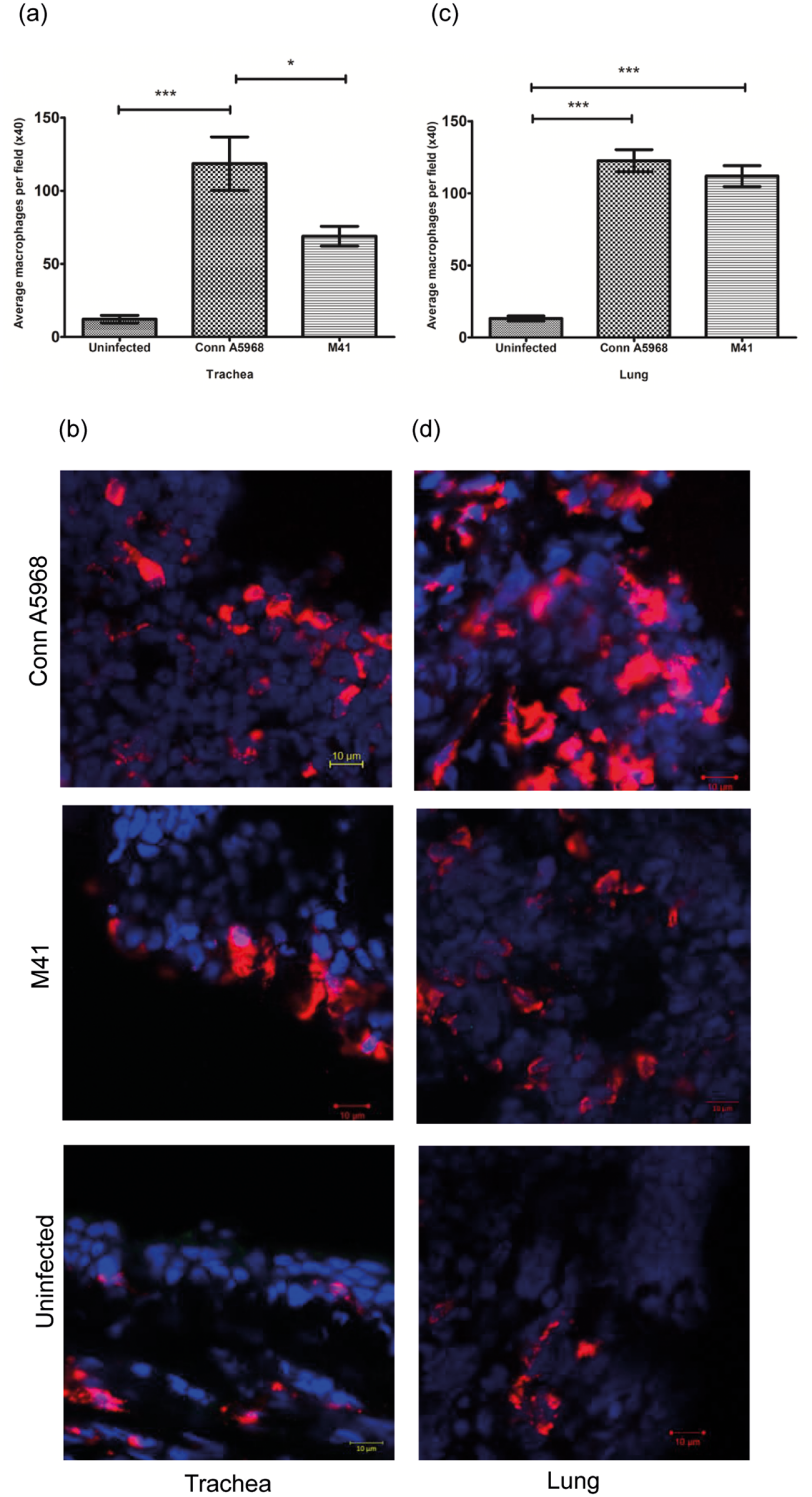
IBV M41 and Conn A5968 infections increase macrophage number in trachea and lungs. Six days old SPF chickens were infected intra-tracheally as described in the materials and methods. At 4 dpi, the trachea and lungs were sampled and preserved in OCT for the quantification of macrophage numbers. The 5μm thick frozen sections were stained for macrophages using mouse anti-chicken macrophage primary antibody followed by the secondary anti-mouse antibody conjugated with Dylight 550^®^. An epifluorescence microscope was used for the quantification of macrophage in immunostained sections. Statistical analysis for identifying group differences was done based on ANOVA test setting the P value <0.05. Trachea and lung macrophage quantitative data, as well as representative immunofluorescent images from each treatment group are given.

The macrophage quantification data and representative immunofluorescent images of trachea and lung of IBV infected and control chickens are illustrated in Fig 2. The macrophage numbers in the IBV M41 infected trachea and lungs were 68.9 ± 14.5 and 111.9 ± 15.1 respectively. At the same time point, in IBV Conn A5968 strain infected chickens 118.6 ± 39.1 and 122.6 ± 16.0 macrophages were present in the trachea and lungs respectively. In contrast, there were only 12.2 ± 7.1 and 13.2 ± 4.92 macrophages present in the control trachea and lungs respectively. There were significantly higher macrophage numbers in both the trachea (Fig 2a and 2b) and lungs (Fig 2c and 2d) of IBV Conn A5968 (P<0.0001) and M41 infected chickens (P<0.01 in trachea and P<0.0001 in lung) when compared to the uninfected controls. In the trachea, higher macrophage numbers were also found in chickens infected with IBV Conn A5968 strain compared to the IBV M41 strain (P<0.01) (Fig 2a and 2b).

### IBV M41 and Conn A5968 strains replicate in macrophages of lungs and trachea *in vivo*

**Fig 3:**
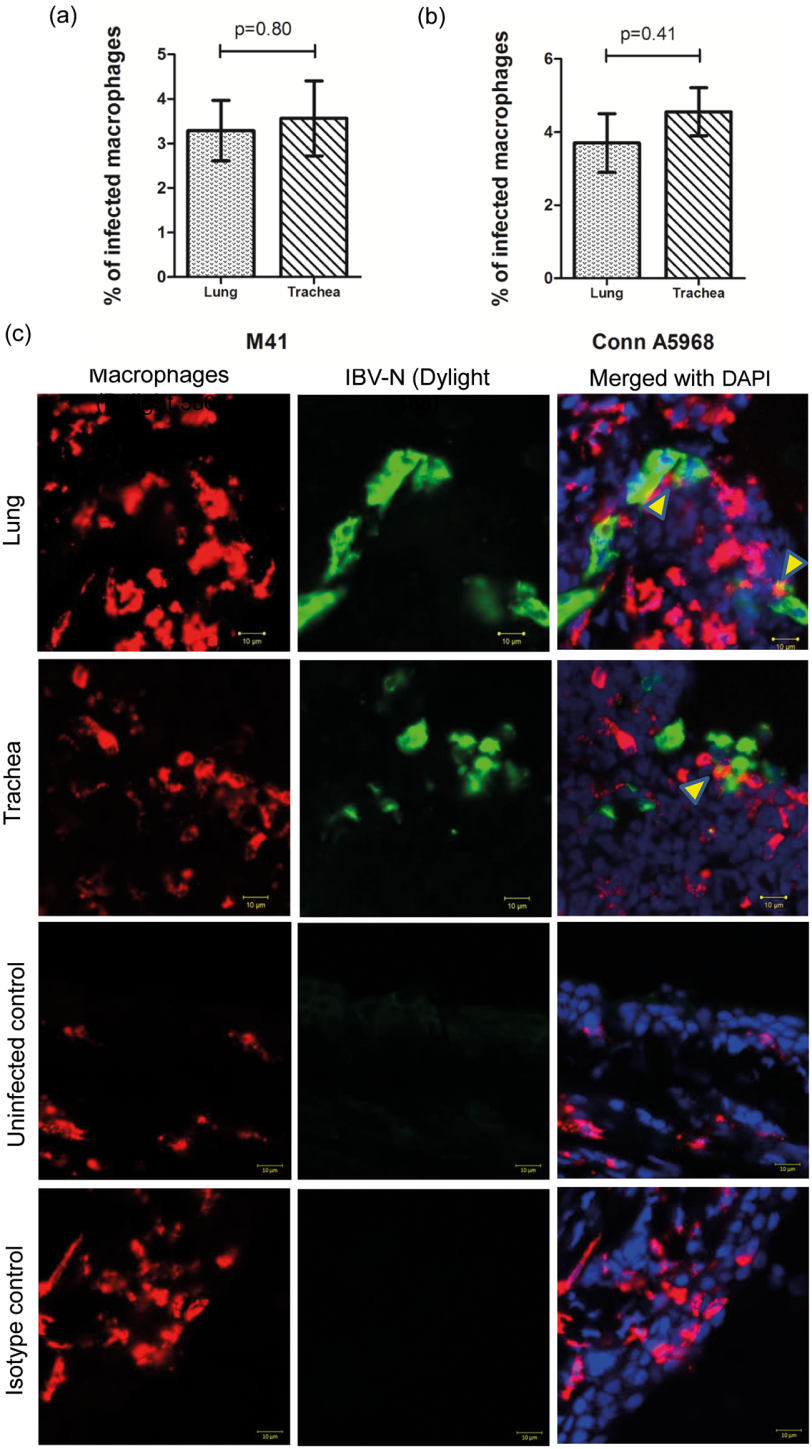
Quantification of IBV M41 and Conn A5968 antigens in macrophages in chickens. Six days old chickens were infected intra-tracheally as indicated in the Fig 1 legend and trachea and lungs were sampled at 4 dpi for double immunostaining of macrophages and IBV antigens. The sections were stained for both macrophages and IBV antigen as has been described in the materials and methods and a confocal microscope was used to obtain representative images. Macrophages were visualized with Dylight 550^®^ (red) and IBV antigens were visualized with Dylight 488^®^ (green). Statistical analysis for identifying group differences was done based on Student’s t test. Nuclei were stained with DAPI (blue). IBV infected macrophages are shown with arrow heads in the merged picture. Both isotype and uninfected controls are shown.

Double immunofluorescent assay revealed that IBV antigens were detectable in 3.6% (±0.8) and 3.3% (±0.7) of trachea and lung macrophages respectively in IBV M41 strain infected chickens (Fig 3a and 3c). In IBV Conn A5968 strain infected chickens, respectively 4.6% (±0.9), and 3.7 (±0.8) of the trachea and lung macrophages carried IBV antigens (Fig 3b and 3c). The number of macrophages carrying IBV antigens in trachea and lung were not significantly different for both IBV M41 and Conn A5968 strains (P>0.05).

### IBV M41 and Conn A5968 strains replicate in macrophages *in vitro* and establish productive infection

**Fig 4:**
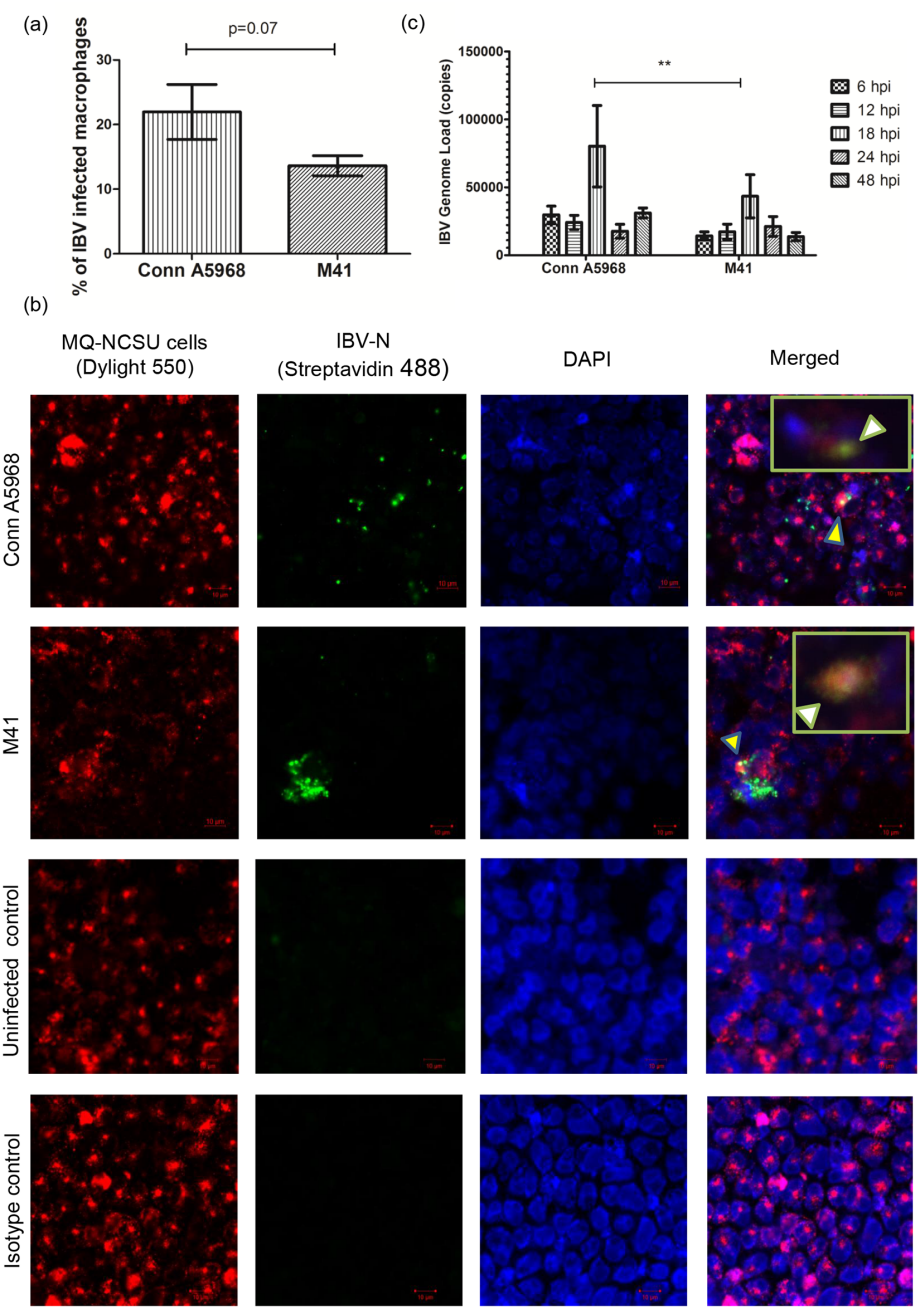
IBV M41 and Conn A5968 strains replicate in macrophages *in vitro* and establish productive infection. 1.5x 10^6^ of MQ-NCSU cells were cultured on plastic coverslips in 6-well plates for 24 hours before infected with 0.1 MOI of IBV Conn A5968 and M41 strains. After 1 hour of adsorption period, inoculum was drained and cells were washed 2 times with HBSS and incubated at 40°C for another 24 hours. At the end of 24 hours, cell membranes of macrophages were stained with horseradish wheat agglutinin conjugated with Alexa Flour 594. Then the cells were fixed using 4% paraformaldehyde, stained for IBV N antigen and visualized using Dylight 488^®^ conjugated secondary antibody and nuclear staining was done with mounting media with DAPI at the time of mounting coverslips on the slides. (a) Percentage of infected macrophages (MQ-NCSU cells) is shown for each IBV strain. (b) Representative confocal microscopy images showing M41 and Conn A5968 IBV antigens in macrophages. (c) The MQ-NCSU cells were cultured on 6-well plates for 24 hours before infected with 0.1 MOI of IBV Conn A5968 or M41 strains. After 1 hour of adsorption period, inoculum was drained and cells were washed 2 times with HBSS to preclude subsequent quantification of the residual IBV inoculum and continued to incubate at 40°C before collecting cell culture supernatant at 6, 12, 18, 24 and 48 hpi for IBV viral RNA concentration determination using a qPCR assay. All the treatments were done in triplicate and qPCR reactions were carried out in triplicate for each sample as well. For each time point, average number of IBV copies per 200 ng of starting RNA of both IBV Conn A5968 and M41 strains are shown with SEM. ANOVA test was performed to identify group differences and the differences were considered significant at P<0.05. The results represent pooled data of three independent experiments.

As indicated by an *in vitro* study 13.6% (± 1.6) and 21.97% (± 4.3) of the macrophages were infected when these cells were infected with IBV M41 and Conn A5968 strains respectively (Fig 4a and 4b). The percentage of infected macrophages did not significantly differ between IBV M41 and Conn A5968 strains (p>0.05).

As an indication of productive IBV Conn A5968 and M41 infections, IBV RNA concentration in culture supernatants of MQ-NCSU cells was measured over the course of the infection *in vitro*. The IBV RNA concentration in the cell culture supernatants peaked at 18 hpi. Thereafter, a gradual decrease in the RNA concentration (Fig 4c) could be observed. Both strains showed a similar pattern but the peak RNA concentration was significantly higher with IBV Conn A5968 strain infection compared to the M41 strain (p<0.001) infection.

### Macrophage infections of IBV M41 and Conn A5968 strains produce coronavirus-like particles within macrophages *in vitro*

**Fig 5:**
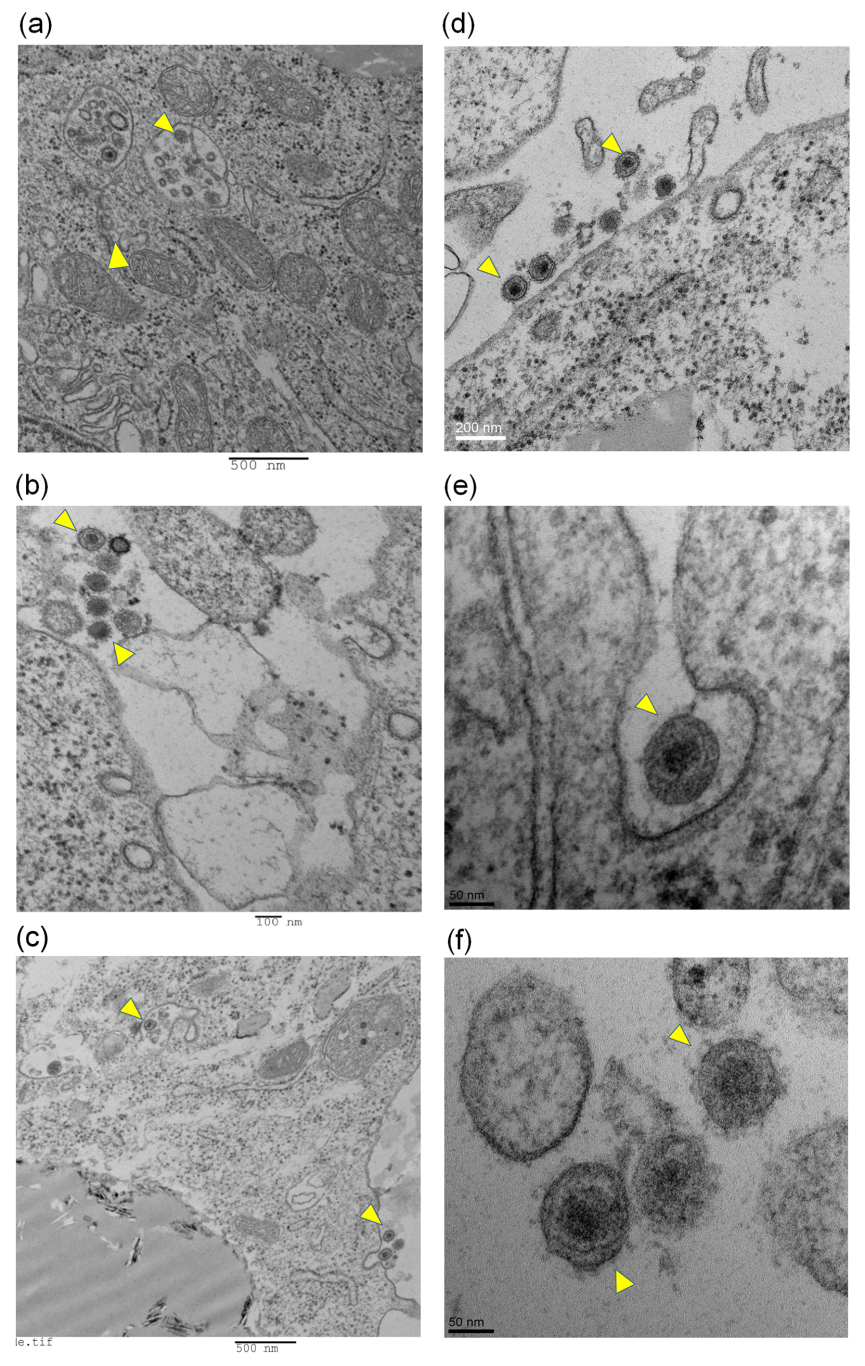
Macrophage infections of IBV M41 and Conn A5968 strains produce coronavirus-like particles within macrophages *in vitro*. MQ-NCSU macrophage cells were cultured on T25 culture flasks until 90% confluence was achieved. Then, the culture media was drained, washed twice with HBSS in order to remove any residual virus inoculum and infected with 0.1 MOI of M41 or Conn A5968 IBV strains. Each experiment was performed in triplicate including an uninfected control. After one hour of adsorption period, the plates were replaced with serum-free 2X MEM media and incubated for further 24 hours at 37°C and 5% CO_2_ before harvesting cells for TEM imaging. Low resolution images (a, b, c) and high resolution images (d, e, f) are shown with coronavirus-like particles (arrowheads). (a), (b) and (d) = M41 strain of IBV; (c), (e) and (f) = Conn A5968 strain of IBV.

TEM images showed that coronavirus-like particles are present both inside and outside of the IBV- infected macrophages (Fig 5). Intracellular particles were observed in vacuoles (Fig 5a and 5c). Under the high resolution TEM the morphology of the viral particles was very similar to the coronavirus morphology (Fig 5d, 5e and 5f). The characteristic spike proteins of coronavirus were visible on the surface along with electron dense nuclear core as has been illustrated in Fig 5e and 5f).

### Replication of M41 and Conn A5968 strains of IBV in macrophages alter selected antimicrobial functions of macrophages

**Fig 6:**
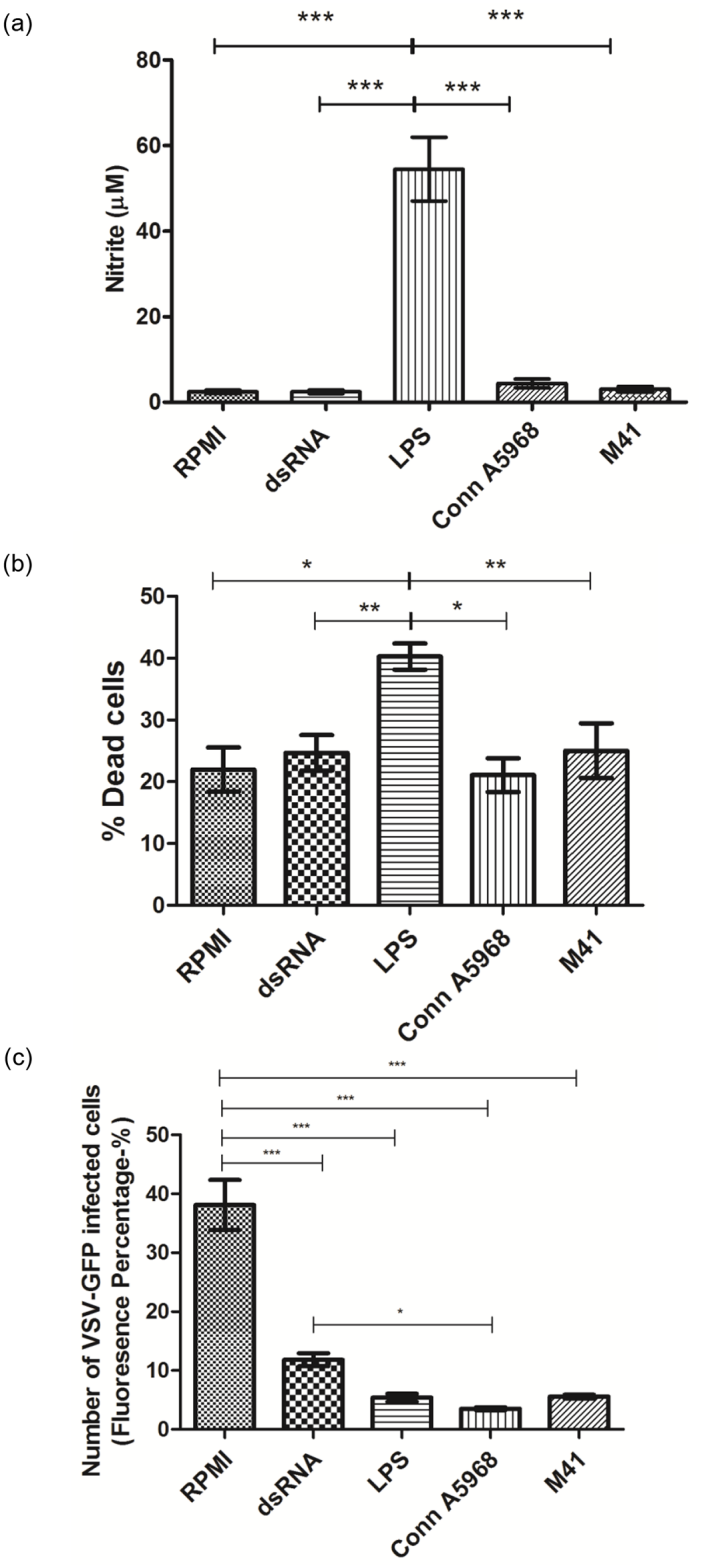
Replication of M41 and Conn A5968 strains of IBV in macrophages reduces NO production but type IFN activity. MQ-NCSU cells (0.75 x 10^6^ per well) were seeded on 12-well plates and either infected or treated with 0.1 MOI of IBV Conn A5968 strain, IBV M41 strain, 1μg/ml LPS (positive control for NO production), 50μg/ml dsRNA (positive control for type 1 IFN activity) or RPMI media (negative control). (a) After 24-hour of stimulation period, cell culture supernatants were collected and NO content in a portion was measured using the Griess assay. Average nitrite contents with each treatment and controls (a) are shown. (b) Following the collection of culture supernatants, the MQ-NCSU cells were scraped and trypan blue dye exclusion method was used to count dead and live cells. The percentage of dead cells with each treatment and controls (b) are given. (c) The remaining portions of macrophage cell culture supernatants were transferred (250μl) to DF-1 cell monolayer for the quantification of type 1 IFN activity. Twenty-four hours following the transfer of culture supernatants, the DF-1 cells were infected with VSV-GFP at the rate of MOI = 0.1. At 24 hours post infection of DF-1 cells, the cells expressing GFP signals were observed under epifluorescent microscope after fixation with 4% paraformaldehyde and nuclear staining with Hoechst 33342 (Image-iT^™^ LIVE Plasma Membrane and Nuclear Labeling Kit (I34406), Invitrogen, Eugene, Oregon, USA). Average fluorescent percentages with each treatment and controls (c) are shown. The results represent pooled data of three independent experiments. * = p< 0.01, ** = p< 0.001, *** = p<0.0001.

The production of NO, in response to infection with M41 and Conn A5968 strains of IBV, appears to be negligible (Fig 6a). In other words, infection of MQ-NCSU cells with M41 and Conn A5968 strains of IBV did not induce NO production. Production of NO was significantly higher in LPS treated group (positive control) than in the untreated control and dsRNA treated and M41 and Conn A5968 strains of IBV infected groups (p<0.0001). As expected we did not observe a higher NO production in the dsRNA treated group (positive control for type 1 IFN activity) when compared to the untreated controls (P>0.05).

As illustrated in the Fig 6b, a difference in dead cell percentage between RPM1 and dsRNA treated and M41 and Conn A5968 strains of IBV infected MQ-NCSU cells was not observed (P>0.05). The percentages of dead cells (approximately 20%) in these groups were significantly lower when compared to that observed following LPS treatment (approximately 40%) of MQ-NCSU cells (p< 0.01 to p< 0.001).

As illustrated in Fig 6c, similar to dsRNA and LPS treatments, replication of both M41 and Conn A5968 strains of IBV in macrophages significantly increased the type 1 IFN activity when compared to the unstimulated controls as indicated by type 1 IFN bio-assay (P<0.0001 respectively). When compared to the positive control, dsRNA, both M41 and Conn A5968 strains of IBV showed higher type 1 IFN activities (P<0.0001). When compared to M41 strain of IBV, Conn A5968 strains of IBV showed a higher type 1 IFN activity (P<0.0001).

## Discussion

We used two strains of IBV, which are known to produce clinical signs and pathology, to investigate whether the macrophages are vulnerable as a site of IBV replication as has been documented for other coronaviruses. We also wanted to determine if these strains interfere with the antimicrobial functions of avian macrophages during the process of replication. To address these questions, we conducted both *in vivo* and *in vitro* experiments. With our initial observations, we verified that a portion of macrophages present in trachea and lungs carry antigens of both M41 and Conn A5968 strains of IBV. IBV M41 strain is known to induce severe clinical signs compared to IBV Conn A5968 strain. Second, we observed that these two strains of IBV can infect macrophages *in vitro* forming fully infectious IBV viral particles and establishing productive replication leading to release of virus. Third, we showed that the replication of M41 and Conn A5968 strains of IBV in macrophages *in vitro* does not induce NO production. Interestingly, we were also could substantiate that the replication of both M41 and Conn A5968 strains of IBV minimally impede the induction of type 1 IFN activity of macrophages.

Of the avian viruses, avian influenza virus [11], infectious laryngotracheitis virus [11], reovirus [11, 16], adenovirus [11], infectious bursal disease virus [11, 17], Newcastle disease virus [11] and Marek’s disease virus [6] infect macrophages playing various roles in the pathogenesis of these viral infections in chickens. The Coronaviridae family including viruses such as Middle Eastern respiratory syndrome (MERS)-CoV [9], SARS corona virus [18], feline infectious peritonitis virus [7], porcine epidemic diarrhea virus [19], porcine transmissible gastroenteritis virus [20] and human respiratory coronavirus [21] are known to replicate in macrophages. Our work provides evidence that some strains of IBV (e.g. M41 and Conn A5968) are also capable of infecting macrophages *in vivo* and *in vitro*.

As evidenced by the *in vivo* work, only 3–4% of macrophages inhabiting the respiratory tract are infected with IBV. *In vitro*, only 13–21% of MQ-NCSU cells carried viral antigen when infected with IBV at the rate of 0.1 MOI. Further, we observed only moderate (2–3 fold) increase in IBV RNA in culture supernatants of MQ-NCSU cells peaking around 18 hours. Although it is possible that higher MOI of IBV may lead to higher increase in IBV RNA in culture supernatants [4], this potential increase of IBV infection in macrophages following higher MOI should be confirmed with further investigations.

With the finding of replication of M41 and Conn A5968 strains in macrophages, further studies are required in order to see whether the IBV established infection in macrophages is strain dependent. In general, many members of the family Coronaviridae target macrophages as a site of viral replication. However, as shown before this was not the case for Beaudette and Massachusetts type 82822 strains of IBV [11]. Although we could not find any information about the pathogenic potential of Massachusetts type 82822 strain of IBV, we are aware that IBV Beaudette strain is a nonpathogenic strain. It is known that the M41 and Conn A5968 strains of IBV we used in our experiments are pathogenic in the field and infect naïve chickens [5, 22] leading to negative consequences on poultry production. It is likely that strain difference and macrophage tropism could be related to difference in virulence. This is in agreement with observations made in studies on feline coronavirus infections [23]. In cats, there are two related corona viral strains and only one of them replicates in macrophages leading to deadly systemic infection referred to as feline infectious peritonitis. The second question arising from our finding relates to the type of entry receptor used by IBV expressed on macrophages. Although dendritic cell-specific intercellular adhesion molecule-3-grabbing non-integrin (DC-SIGN) in macrophages has been identified as a receptor involved in recognizing carbohydrate components in pathogens, influenza virus is capable of using this receptor for entry into macrophages [24]. DC-SIGN also has been shown as an entry receptor for feline infectious peritonitis corona virus infection of monocytes [25]. Recently, it has also been shown that artificial expression of DC-SIGN on IBV resistant cells could make these resistant cells vulnerable to certain IBV strains including M41 and Conn strains [26]. However, further investigations are required to identify an entry receptor for IBV infection of macrophages. DC-SIGN molecules are potentially expressed on avian macrophages for recognizing pathogen associated molecular patterns consisting of carbohydrate moieties [27].

Avian macrophages are known to produce antiviral molecules such as NO and type 1 IFN [28, 29]. These two immune mediators are well known for their innate host responses against coronaviruses such as SARS-CoV [30–32]. Whether IBV replication is inhibited by NO originating from macrophages is not known but some other avian viruses such as infectious laryngotracheitis virus [33] and reovirus [34] can be inhibited by NO originated from avian macrophages. In addition, avian viruses such as avian paramyxovirus can be inhibited by type IFNs [35]. With this rationale, we directed our focus to evaluate NO production and type 1 IFN activity of macrophages during the IBV replication in avian macrophages. Since we show that avian macrophages are vulnerable to M41 and Conn A5968 IBV infections, we questioned whether IBV M41 and Conn A5968 strains could impair the pathways leading to production of NO and type 1 IFN activity while they replicate within macrophages. First, we found that IBV M41 and Conn A5968 infections of macrophages do not lead to the production of NO *in vitro*. This observation is in agreement with the observation made in murine macrophages using H5N1 and H1N1 influenza viruses [36], swine macrophages using classical swine fever virus [37] and avian macrophages using infectious bursal disease virus (IBDV) [38] infections. The latter study in avian macrophages showed that certain strains of IBDV inhibit NO production. Second, we found that IBV M41 and Conn A5968 infections of macrophages do not interfere with the type 1 IFN activity *in vitro* although inhibition of type 1 IFN production has been shown by other corona viruses such as porcine epidemic diarrhea virus [39], porcine delta coronavirus [40], SARS-CoV [41] and murine coronavirus [42]. Although, IBV has not been linked to inhibition of type 1 IFN activity in macrophages as observed currently, it has been shown to inhibit type 1 IFN response in chicken epithelial and fibroblast cells [43–45]. Although it has been shown that IBV accessory protein 5b is responsible for this down regulation of type 1 IFN activity *via* the inhibition of signal transducer and activator of transcription (STAT)1 in epithelial and fibroblast cells [44, 45], further studies are required to investigate why accessary 5b protein of IBV is not regulating the type 1 IFN activity in macrophages.

In the current study, we observed that M41 and Conn A5968 strains of IBV replicate in macrophages *in vivo*. However, we could not extend our observations further to elucidate the consequences of IBV-macrophage interaction in chickens. Although M41 and Conn A5968 strains of IBV infections first establish infections in the respiratory tract, these two strains of IBV are known to replicate in a variety of tissues leading to pathology in these tissues [4]. For example, M41 strain of IBV replicates in shell gland, kidney and tissues of gastrointestinal tract in addition to the respiratory tract [3, 46]. Similarly, IBV Conn A5968 strain has been shown to be present in bursa of Fabricius, intestine and respiratory tract [46]. The ability of IBV to replicate in macrophages can be a major determinant of pathogenicity as it may facilitate carriage to susceptible tissues and organs as demonstrated in some other viral infections such as Ebola virus [47], demyelinating strains of Theiler's murine encephalomyelitis virus [48] and HIV [8]. The ability to infect macrophages can also determine the severity of some corona viral infections such as feline infectious peritonitis virus [49] and MERS-CoV infections [50]. Similar to other host-viral interactions, whether IBV replication in avian macrophages are also results in relaying the virus to other tissues with a consequence of enhanced IBV pathogenicity is unknown and this is a subject of further experiments.

As illustrated in the Fig 6b, we observed approximately 20% of dead MQ-NCSU cells following 24 hours in the culture even without a treatment or infection. This is difficult to explain but it is possible that the process of dislodging cells for enumeration may have killed some cells. It is also important to note that without a treatment or infection there was a basal level of NO production (Fig 6b) and NO could decrease the cell survival. In agreement with this view, it has been shown that NO is capable of inducing apoptosis of mouse macrophages [51]. In the current study, we also noted an association between LPS induced NO production (approximately 60μM) in MQ-NCSU cells and % dead cells (approximately 40%) (Fig 6b).

It is important to note that most of the IBV-stained cells were epithelial cells and only 3–4% of the macrophages contained IBV antigen as indicated by overlapping fluorescent signals. In lung and trachea sections, there is a possibility of false positive co-localization due to fluorescent signals originating from two or more overlapping cells rather than fluorescent signals originating from a single cell. This potential false positive co-localization of fluorescent signals was minimized in a number of ways. First, lung and trachea sections were cut at 5μm thickness which is substantially smaller than the size of macrophage and epithelia cells. Second, we used a double immunostaining technique to stain both macrophages and IBV antigens sequentially in a single section. These double immunostained sections were observed in two separate channels and then merged to visualize the co-localization. Third, we confirmed the *in vivo* observations of co-localization of IBV and macrophage antigens *in vitro* using IBV infected monolayers of macrophages. Use of macrophage monolayers precluded the possibility of false positive co-localization of IBV and macrophage antigens due to overlapping cells *in vitro*.

In conclusion, our data show evidence for the fact that avian macrophages support IBV replication *in vivo* and *in vitro*. It was also noted that the process of IBV replication in macrophages inhibits the NO production but not the type 1 IFN activity of macrophages. Further studies are required to determine the significance of avian macrophage-IBV interactions in the pathogenesis of IBV infection in chickens.
